# Boron-Rich Biologics Enabled by Reactive Organic Carboranes

**DOI:** 10.1021/jacsau.6c00410

**Published:** 2026-05-20

**Authors:** Anže Jenko, Urban Barbič, Aljaž Renko, Ching-Pei Hsu, Dane Jemc, Špela Makuc, Lana Jamnik, Gregor Marolt, Vera Župunski, Andrei Loas, Bradley L. Pentelute, Martin Gazvoda

**Affiliations:** † 37663 University of Ljubljana, Faculty of Chemistry and Chemical Technology, Department of Chemistry and Biochemistry, Večna pot 113, Ljubljana 1000, Slovenia; ‡ 2167 Massachusetts Institute of Technology, Department of Chemistry, 77 Massachusetts Avenue, Cambridge, Massachusetts 02139, United States; § The Koch Institute for Integrative Cancer Research, Massachusetts Institute of Technology, 500 Main Street, Cambridge, Massachusetts 02142, United States; ∥ Center for Environmental Health Sciences, Massachusetts Institute of Technology, 77 Massachusetts Avenue, Cambridge, Massachusetts 02139, United States

**Keywords:** carborane, bioconjugation, NHS esters, peptides, antibodies, hybrid polymers, cancer, BNCT

## Abstract

Carboranes are endowed with unique structural and electronic features and are applied across diverse research areas. General methods for their high-content incorporation, however, remain limited. We report the development of organic carborane reagents based on *N*-hydroxysuccinimide (NHS) esters as a versatile platform for selective conjugation to amine-containing residues of small molecules and polypeptides. Bifunctional carborane reagents obtained by this method enable peptide-driven multimerization into carborane–peptide polymers, an original class of hybrid materials. Therapeutic antibodies functionalized with these reagents yield boron-rich conjugates containing up to 13 carboranes per antibody molecule that retain native antibody activity. Linker length is critical for maximizing loading efficiency. Mass spectrometry mapping revealed up to 30 preferential lysine modification sites per antibody, located outside the complementarity-determining region(s). Prevalence-ranked lysine mapping of IgG antibodies under native-like conditions using NHS esters bearing hydrophobic (carborane) substituents provides a useful framework for the rational design and optimization of antibody–drug conjugates. These constructs, integrating therapeutic antibody targeting features with boron delivery capabilities, hold promise as multimodal agents for boron neutron capture therapy (BNCT).

## Graphical abstract

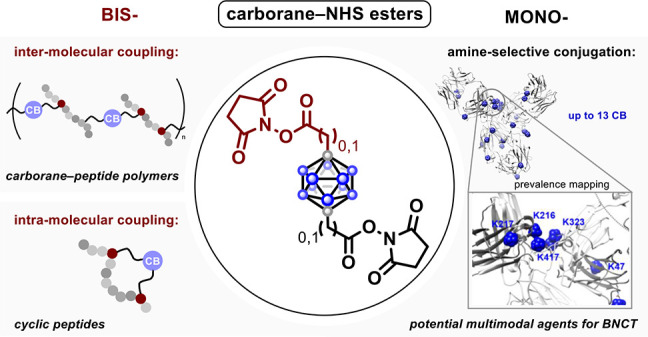

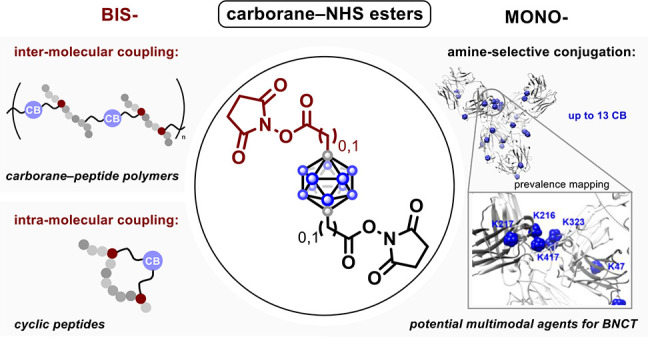

## Introduction

Carboranes are boron-rich clusters endowed with unique stability and electronic properties that underpin applications ranging from carbocyclic mimics in medicinal chemistry and functional motifs in polymers and metal–organic frameworks to electron reservoirs in catalysis and boron carriers for neutron capture therapy (BNCT) (Figure 1a).^1–3^ Despite their broad potential, functionalization of the carborane cage is synthetically demanding,^1,2,4–7^ and their integration into structurally complex targets, such as biomolecules, presents further challenges.^8,9^ For example, peptide and protein carborane conjugates promise precise boron delivery for BNCT, but overcoming synthetic barriers is key to unlocking their full potential.^10–13^

**Figure 1:**
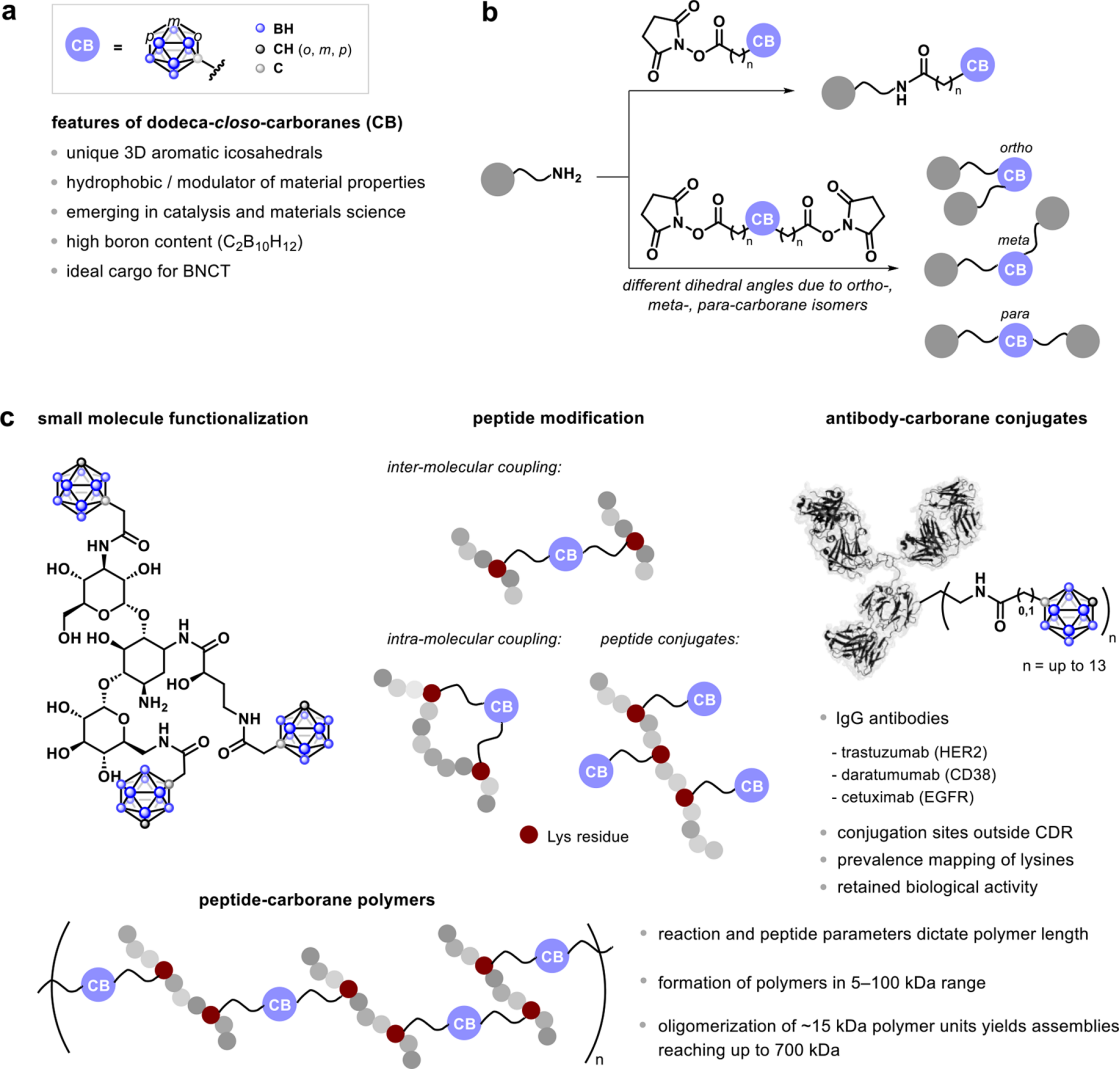
Structures and features of (bio)­molecules produced within this study. (a) Structure and general features of carboranes (dodeca-*closo*-carboranes). (b) Reactions of carborane–*N*-hydroxysuccinimide (NHS) esters enable functionalization of diverse structures through amine-selective reactions. Additional NHS ester moieties on carborane, forming bifunctional carborane–NHS esters, allow inter- and intramolecular coupling, with substituent orientation depending on the carborane type (*ortho*, *meta*, and *para*). (c) Developed reagents enable carborane introduction into small molecules, peptide functionalization (inter/intra-molecular coupling), and antibody conjugation. Reagents allow installation of up to 13 carboranes per antibody, with conjugation occurring on lysine residues outside CDR regions, as determined by nanoscale liquid chromatography-tandem mass spectrometry (nLC–MS/MS), indicating prevalence-ranked lysine reactivity under the studied conditions, while the antibodies retain their activity in cell-based assays. Under appropriate conditions, bifunctional carborane–NHS esters with peptides containing two Lys residues undergo polymerization, yielding polymer chains ranging from 10 kDa to 100 kDa, depending on the structure of the monomers and reaction conditions.

Current strategies for carborane incorporation into biomolecules largely rely on solid-phase peptide synthesis using carborane-bearing noncanonical amino acids or late-stage acylation, both of which are hampered by side reactions, such as deboronation during Fmoc removal.^14^ Alternative methods, including ribosomal incorporation of l-carboranylalanine,^15^ cysteine borylation with Pt­(II) organometallic reagents,^16^ or multistep conjugations to oxidized proteins,^17,18^ have expanded the toolbox; however, efficient late-stage modification of peptides and proteins in aqueous media remains scarce.

Beyond bioconjugation, carboranes have also been embedded into polymers,^19^ metal–organic frameworks (MOFs),^20^ and catalysts,^21^ wherein they impart unique structural and electronic properties. These advances, however, often rely on specialized and case-specific syntheses. General and versatile methods for introducing carboranes into biomolecules and materials would therefore be highly valuable, both for advancing BNCT and for unlocking new opportunities in catalysis and materials design.

Following recent palladium-mediated strategies for carborane installation onto small molecules, peptides, and proteins based on the selective reactivity of thiols (cysteine) with palladium oxidative addition complexes,^22^ we sought to develop a complementary approach targeting amine-containing groups. Amines are ubiquitous in small molecules and in the lysine side chains of peptides and proteins, making them attractive targets for functionalization. Among established reagents for amine-selective chemistry, *N*-hydroxysuccinimide (NHS) esters are especially versatile.^23,24^ To our knowledge, however, carborane–NHS esters have not yet been reported.

Here, we describe the development of carboranes functionalized with NHS esters (Figure 1b) and demonstrate their broad application in modifying small molecules, peptides, and proteins (antibodies) (Figure 1c). Notably, bis-NHS carboranes can act as interlinking fragments, enabling both inter- and intramolecular peptide coupling, the latter of which can, for example, yield peptides with hydrophobic motifs. Moreover, under suitable conditions, these reagents drive the formation of carborane–peptide polymers, an original class of materials composed of structurally and chemically distinct fragments: hydrophobic carborane clusters and hydrophilic peptide domains (Figure 1c).

## Results and Discussion

We devised the synthetic methodology for producing carborane–NHS ester reagents **1**–**7** (Figure 2). Carboxylic acid-functionalized carborane **8** was converted to the corresponding NHS ester **1** and isolated in 14% yield, owing to its lower stability (Figure 2a). Following modified literature procedures,^25^ carboxylic acids of *meta*- and *para*-carboranes were accessed, and their subsequent reactions afforded *meta*- and *para*-carborane–NHS reagents **2** and **3** in 50% and 87% yield, respectively (Figure 2b). In contrast to **1**, structures **2** and **3** have a methylene linker between the carbonyl and NHS group, providing greater steric flexibility, which proved to be important for further bioconjugation reactions.

**Figure 2:**
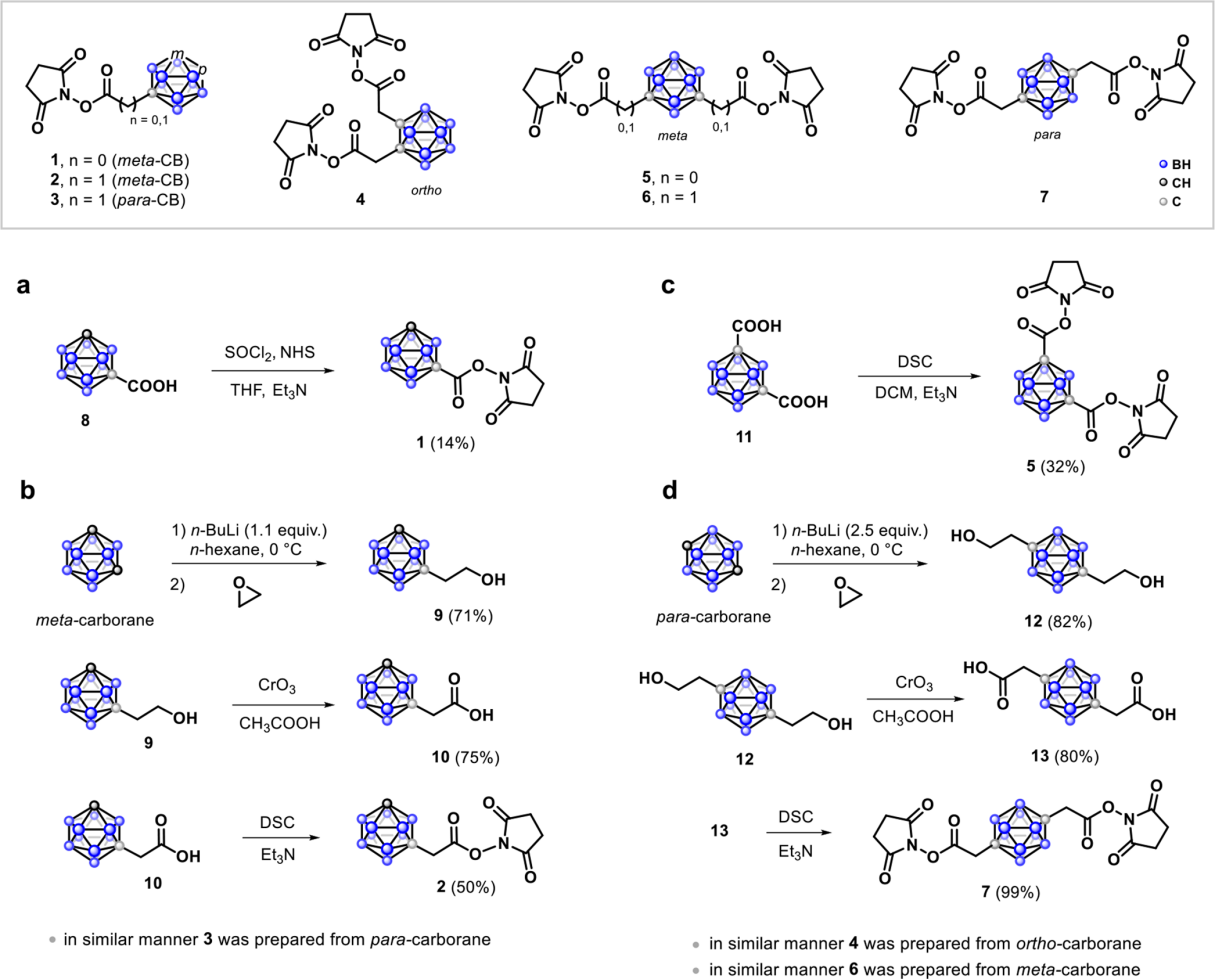
Structure and derivatization of carborane–NHS esters. Synthetic pathways for the preparation of (a, b) mono-NHS-functionalized carboranes **1**–**3** and (c, d) carboranes functionalized with two NHS ester groups **4**–**7** are shown. Isolated yields are indicated in parentheses. For more details on the synthesis of **3**, **4**, and **6**, see the Supporting Information, Section 2.1.

To further increase the functionalization potential of these structures, we prepared reagents with two NHS esters per carborane molecule **4**–**7**, which are suitable for both inter- and intramolecular coupling (Figure 2). In monofunctional carborane–NHS esters **1**–**3**, the isomer mainly affects cage stability, whereas in bifunctional systems, it also dictates the orientation of reactive groups. Given the 3D nature of carboranes (overall diameter of the icosahedral cage ∼5–6 Å; ∼1.5 times larger than benzene),^1–3^ controlling substituent position could be important. We therefore prepared *ortho*-, *meta*-, and *para*-carborane–bis-NHS esters **4**–**7**.

We synthesized NHS esters of *ortho*-, *meta*-, and *para*-carborane **4**, **6**, and **7**, in which the NHS ester is attached to the carborane via a methylene linker. Additionally, we prepared the bifunctional reagent of the *meta*-analog with two NHS esters directly attached to carborane **5**. The latter was prepared from dicarboxylic acid **11** in 32% isolated yield (Figure 2c). The methylene-linked structures were synthesized analogously to **2** but using excess *n*-BuLi and oxirane reagents to generate the corresponding alcohols, e.g., **12**, which were subsequently converted to bis-NHS esters, such as the *para*-carborane derivative **7** (Figure 2d). In a similar manner, reagents **4** and **6** were obtained from *ortho*- and *meta*-carboranes, respectively. Reagents **1**–**7** were sufficiently stable for handling and short-term analysis under ambient conditions (Figure S1); however, long-term storage was best achieved under an inert atmosphere at −20 °C.

Both types of reagents, bearing either one (Figure 3a) or two NHS groups (Figure 3b), proved suitable for amine-targeted reactions with small molecules and peptides. Reactions of amine-bearing small molecules and peptides with these reagents afforded carborane-functionalized products in nearly quantitative conversion, as determined by ^1^H NMR and LC–MS analyses (see Sections 2.1.and 2.2., and Figures S2–S26 in Supporting Information). The corresponding small-molecule-functionalized carboranes were subsequently isolated in 22–89% yields. The lower isolated yields in some cases are attributed to challenging purification. In certain cases, both highly polar carborane-functionalized small molecules, e.g., glycosides, and moderately polar substrates exhibited chromatographic behavior similar to either liberated *N*-hydroxysuccinimide or residual carborane–NHS esters and their corresponding acids, arising from slight reagent excess, thereby complicating separation and lowering isolated yields. In addition, peptide conjugates are well-known to incur material losses during chromatographic purification,^26^ consistent with our observations. Nevertheless, in all cases, chromatographic purification afforded the target products in analytically pure form.

**Figure 3:**
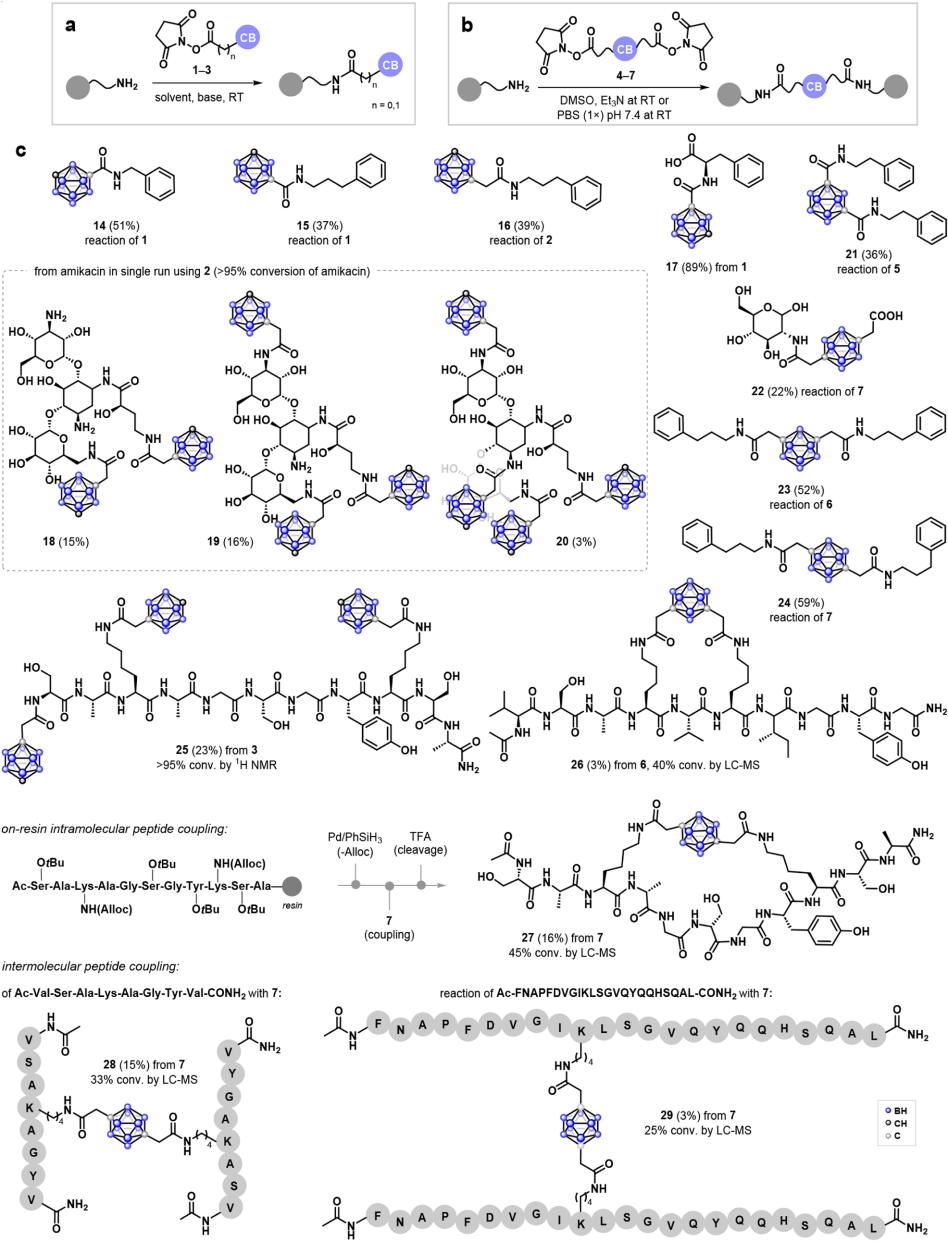
Reactions of amine groups of small molecules and peptides with carborane (a) mono- and (b) bifunctional NHS esters **1**–**7**. (c) Substrate scope of carborane-NHS ester reactivity, yielding boron-rich small molecules and peptides, including intermolecular and intramolecular peptide coupling, the latter having the potential to yield cyclic peptides. Isolated yields are indicated in parentheses.

Reactions of **1** with benzylamine, 3-phenylpropylamine, and d-phenylalanine resulted in products **14**, **15**, and **17** in 59%, 38%, and 89% isolated yields, respectively (Figure 3c). In a similar way, the reaction of **2** with 3-phenylpropylamine afforded **16** in quantitative conversion, as judged by ^1^H NMR, and 39% isolated yield. To demonstrate the ability of the developed reagents to reliably functionalize complex small molecules for biological evaluation, four equivalents of reagent **2** were reacted with amikacin, a clinically used antibiotic,^27^ affording near-quantitative conversion to amikacin derivatives bearing two to four carborane units (**18**–**20**). Despite modest isolated yields for the individual carborane-functionalized amikacin analogues, the reaction enables rapid access to three structurally distinct derivatives in a single step, still providing adequate material for potential downstream biological or catalytic studies. This result highlights the suitability of the platform for late-stage functionalization and diversification of structurally complex bioactive molecules (see sections 2, 4, 5 and 6 in the Supporting Information).

Reactions of the bis-NHS carboranes **5**, **6**, and **7** with 2-phenylethylamine or 3-phenylpropylamine afforded the bridged products **21**, **23**, and **24** in 36%, 52%, and 59% isolated yields, respectively, with the carborane linking the two molecules (Figure 3c). The reaction of **7** with glucosamine under basic conditions gave **22** in 22% isolated yield, with one NHS ester reacting with glucosamine and the other hydrolyzing in the basic medium (Supporting Information, Figure S10, Section 2.1).

Treatment of peptide H-Ser-Ala-Lys-Ala-Gly-Ser-Gly-Tyr-Lys-Ser-Ala-CONH_2_ (64 mM), bearing two Lys residues and a free N-terminal amine, with 10 equivalents of **3** afforded conjugate **25** in quantitative conversion as judged by ^1^H NMR (Figure S11), with 23% isolated yield. We investigated the intramolecular linkage of two lysine residues within the N-terminally acetylated Ac-Val-Ser-Ala-Lys-Val-Lys-Ile-Gly-Tyr-Gly-CONH_2_ peptide to generate a carborane hydrophobic bridge, thereby accessing intrachain carborane-cyclized peptides. At a 14 mM concentration, the reaction gave about 40% conversion into **26** as determined by LC–MS (Figures S12-S13), and the target product was isolated in 3% yield to confirm its structure (Figures S14-S15).

To improve the yield of the intracyclization and to access larger macrocyclic peptides, we pursued an on-resin coupling strategy.^28^ The resin-bound peptide Ac-Ser-Ala-Lys-Ala-Gly-Ser-Gly-Tyr-Lys-Ser-Ala-CONH–resin was prepared by solid-phase peptide synthesis (SPPS) using Alloc-protected lysine side chains. Subsequent Pd/PhSiH_3_-mediated Alloc deprotection provided two free amino groups on resin. Subsequent overnight reaction with reagent **7**, followed by cleavage and global deprotection, afforded compound **27** in 45% conversion (LC–MS) and 16% isolated yield (Figures S16–S19). Further studies are underway to evaluate how Lys spacing in the peptide and the positioning of the NHS groups (*ortho*, *meta*, or *para*) within the carborane reagent influence the efficiency of intramolecular coupling.

Reactions of the *N*-acetylated 8-mer Ac-VSAKAGYV-CONH_2_ (144 mM) and *N*-acetylated obestatin, a ghrelin-derived peptide implicated in the modulation of feeding rhythm and hunger timing (45 mM), were carried out with reagent **7** in DMSO in the presence of Et_3_N. Both peptides contain a single lysine residue and afforded the intermolecular coupling products **28** and **29** in 33% and 25% conversion, respectively, as determined by LC–MS (Figure 3c, Figures S20–S26).

Together, the functionalization results demonstrate that the developed reagents enable efficient modification of structurally diverse small molecules, e.g., carborane–glycoside and amikacin derivatives, thereby expanding access to biologically relevant scaffolds amenable to evaluation in medicinal chemistry and BNCT applications, where carboranes can function as nonpolar, boron-rich carbocyclic framework mimetics (Figure 3).^2,3,8,9^ The successful preparation of peptide–carborane conjugates, including obestatin derivatives and cyclic peptides via bifunctional reagents, further highlights the platform’s utility in potentially enhancing serum stability,^16^ target binding,^15^ and structural features^8,9^ through carborane incorporation.

Carborane-based polymers, MOFs, and catalytic systems are emerging as a new class of functional materials, where the unique 3D geometry, hydrophobicity, and electronic richness of the cage impart properties that cannot be attained with conventional organic or inorganic motifs. From electron reservoir catalysts, ultrastable polyimides, and ROMP-derived copolymers to water-stable, gas-selective MOFs, these architectures showcase the growing potential of carboranes to redefine the design landscape of advanced materials.^29,30^ Finally, boron (carborane)-rich polymers are also discussed as promising candidates for BNCT.^31–33^

While exploring the intramolecular cyclization reactions to **26**, we observed the formation of polymer-like byproducts. This suggested that, under appropriate conditions, larger polymers composed of two structurally distinct fragments, i.e., a carborane and a peptide, can be constructed (Figure 4). To our knowledge, such hybrid architectures are unprecedented and represent first-in-class materials. These structures could hold promise for BNCT, as their composition and tunable architecture may allow for efficient boron incorporation while retaining a good aqueous solubility.

**Figure 4:**
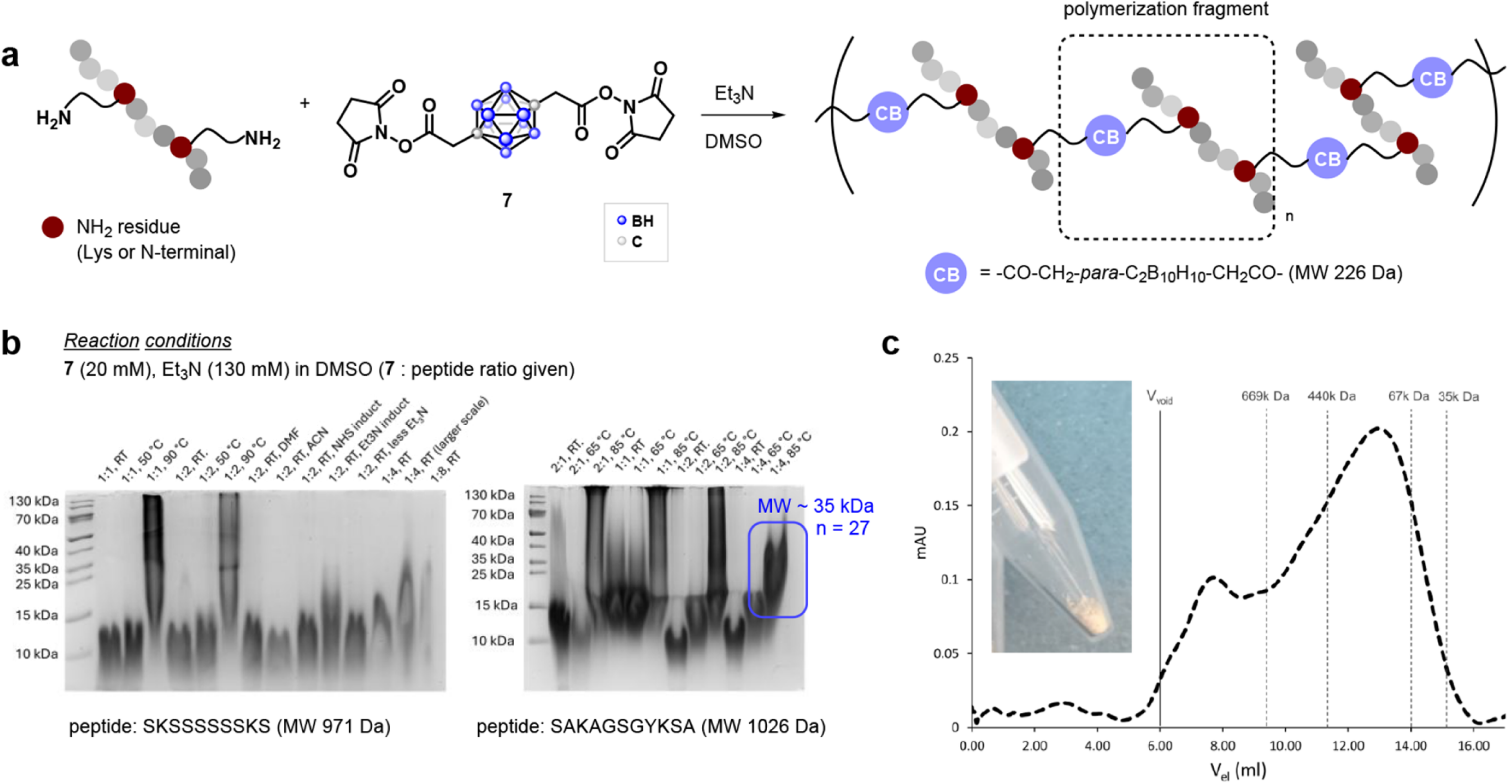
Carborane-NHS reagents can be used to form peptide-carborane polymers. (a) Reaction scheme to construct peptide–carborane polymers. (b) Polymer length is tunable by reaction conditions and peptide structure. Screening reactions were performed on a small scale using 0.4 μmol of the limiting reagent (typically a 20 μL reaction with 20 mM of **7** as the limiting reagent). Prior to gel analysis, the compounds were incubated at 90 °C to disrupt any potential noncovalent interactions. Smearing of bands in the gel is most likely due to sample heterogeneity arising from polymer formation of varying lengths, as shown in Figure S27. (c) Photo of an isolated carborane–peptide polymer, with a size-exclusion (SEC) chromatogram using Superdex 200 Increase 10/300 GL column (Cytiva) showing that the monomer polymer units undergo oligomerization in solution. The results indicate dynamic connectivity, with oligomers reaching up to ∼700 kDa (approximately 45 monomer units of about 15 kDa each). Most of the species, however, fall within the range from ∼70 kDa to ∼440 kDa, corresponding to oligomers of 5–30 monomer units. Approximate elution positions of structures with different molecular weights are indicated, as determined by external standard analysis for Superdex 200 Increase 10/300 GL column (Cytiva) (Figure S33).

To investigate polymer formation, we employed two short model peptides, each containing two Lys residues and a free terminal amino group, and examined how the ratio of bis-NHS carborane reagent **7** to peptide influenced the outcome (Figure 4a, Figures S27–S32, Table S1). Optimization studies (Tables S2–S3) were carried out by systematically varying the reaction concentration (40–320 mM), reagent ratios, solvent (DMSO, DMF, or acetonitrile), temperature (22–90 °C), and the order of reagent addition. Generally, reagent Et_3_N was added last to the mixture of peptide and **7**; however, we also investigated reactions in which **7** was introduced as the final reagent to initiate polymerization, to gain further insight into the polymerization process (Figure 4b). Higher temperatures and larger excess of peptide relative to **7** favored the formation of longer polymers (Figure 4b). The peptide sequence itself also influences the process; for example, while peptides of comparable length, such as SKSSSSSSKS and SAKAGSGYKSA, were both reactive, the latter reacted more efficiently, yielding polymers up to ∼35 kDa, corresponding to about 54 coupling events (*n* = 27). Under elevated temperature and near-equimolar peptide-to-**7** ratios, even longer polymers could be detected, although their formation was less controlled and produced heterogeneous mixtures (Figure 4b).

The formed carborane–peptide polymers were stable under reducing conditions (90 °C in the presence of dithiothreitol (DTT)), to which they were subjected prior to sodium dodecyl sulfate (SDS) polyacrylamide gel electrophoresis (PAGE) analysis (Figure 4b). Since the peptide and carborane units that comprise these polymers can each oligomerize and/or induce oligomerization/aggregation independently,^34–39^ we investigated whether the hybrid architectures preserve this behavior. Size-exclusion chromatography (SEC) revealed the formation of large oligomers of monomer units with molecular weights of up to approximately 700 kDa (about 45 polymer units of 15 kDa), with the majority of oligomers exhibiting molecular weights between 67 kDa and 440 kDa (oligomers of 5–30 polymer units).

We next evaluated the carborane–NHS reagents for antibody conjugation, a transformation that should be performed in almost fully aqueous media at moderate pH and temperature and low-μM antibody concentrations, to assess whether the reagents can transfer the carborane cage onto antibodies under these stringent conditions. IgG antibodies such as trastuzumab, cetuximab, and daratumumab contain approximately 85 lysine residues, of which about 40 are typically modifiable, including some located within complementarity-determining regions (CDRs) that are critical for activity.^40^ Thus, in addition to overall conjugation efficiency, we were interested in determining whether the reagents targeted CDR or non-CDR lysine side chains. Trastuzumab was chosen as a model antibody, while NHS ester **1**, featuring a rigid structure, and reagent **2**, containing a methylene linker between the carborane cage and NHS ester that imparts greater flexibility, served as model reagents. Reactions were carried out at 10 μM antibody concentration in phosphate-buffered saline (PBS) buffer (1×, pH 7.4) at room temperature, with the NHS reagents dissolved in DMSO to address their low aqueous solubility, resulting in a final DMSO concentration of 5% to maintain protein stability (Figure 5a, also Figures S34–S41).

**Figure 5:**
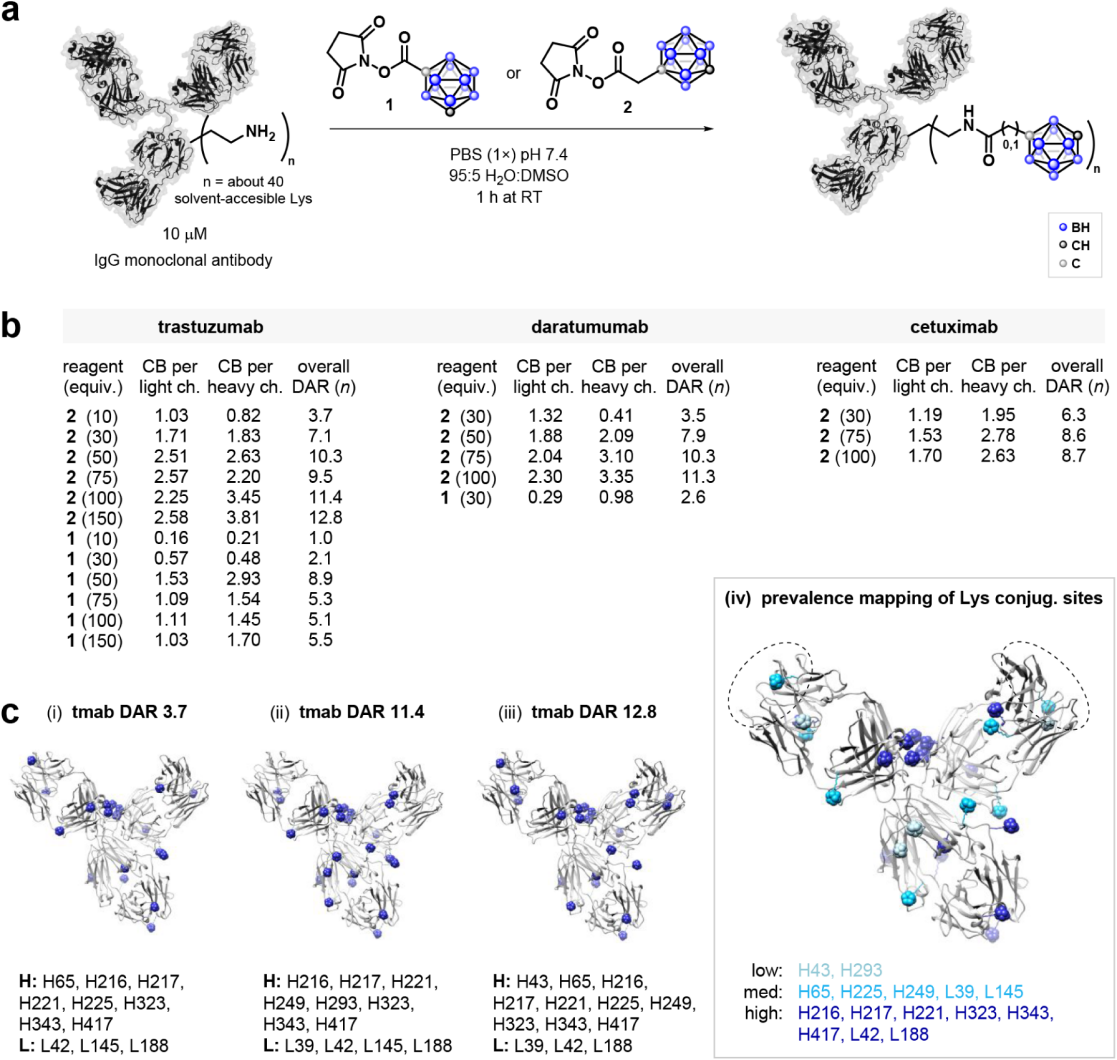
Carborane-NHS reagents efficiently conjugate solvent-exposed lysine side chains of antibodies. (a) Synthetic scheme depicting the conjugation of carborane–NHS reagents **1** and **2** to antibodies. (b) Screening reaction conditions in relation to conjugation of light and heavy chains and overall DAR (drug-to-antibody ratio) according to LC–MS. (c) Mapping of modification sites as determined by nLC–MS/MS in trastuzumab with DAR (i) 3.7, (ii) 11.4, and (iii) 12.8, along with (iv) prevalence of Lys conjugation sites based on statistical analysis of these results. Spherical blue shapes indicate carborane conjugation sites. The color scale in (iv), progressing from light to dark blue, reflects the relative prevalence of modification at each site. Prevalence was determined according to the number of reaction conditions (10, 100, and 150 equiv. of reagent) under which a given site was identified as modified. Sites consistently modified across all three conditions are depicted in the darkest blue shade, whereas those observed only under a single condition are represented in the lightest blue shade. CDR regions are indicated with dashed circles (H: heavy chain, L: light chain; according to the sequence annotation used, H65 corresponds to heavy-chain lysine 65, also described in the literature as HC K65; CB: *meta*-carborane).

Conjugation with reagent **2** enabled the installation of up to 13 carboranes per antibody (Figure 5b). For example, 10, 30, and 50–100 equivalents of **2** yielded average DARs of 3, 7, and 9–11, respectively, while a maximum DAR (drug-to-antibody ratio) of 12.8 was achieved when using 150 equivalents. Although yields were generally high (>50%), antibody recovery dropped by about 50% at the highest loading, and complete precipitation was observed when 200 equivalents of compound **2** were used, indicating the limit of loading under these conditions (see Section 2.4.1. in Supporting Information). Reagent **1** proved less efficient, reaching a maximum DAR of ∼8 at 50 equivalents, while higher loadings led to precipitation and reduced overall conjugation efficiency.

These observations are consistent with literature reports showing that DAR approaches a plateau at higher loading, as commonly observed for highly loaded antibody conjugates.^40,41^ In our system, reagent **2** enabled DAR values of up to 13, whereas the more rigid reagent **1** reached only a DAR of 8. We attribute this behavior primarily to the hydrophobic nature of the carborane moiety, which likely reduces solubility and promotes aggregation at higher loading, while the more rigid incorporation introduced by reagent **1** may leave the carborane(s) more exposed and less conformationally adaptable, further reducing solubility and limiting the attainable DAR.

LC–MS analysis of reduced antibodies showed DAR values ranging from 1.0–2.6 for light chains and 0.8–3.8 for heavy chains (Figure 5b, Tables S4–S5). Similar trends were observed for daratumumab and cetuximab, illustrating that NHS ester **2** consistently delivers relatively high DARs across the studied scaffolds, underscoring its potential for generating boron-rich antibodies.

As antibody–drug conjugates (ADCs) gain increasing clinical prominence,^42^ rigorous conjugation-site analysis is essential for defining structural heterogeneity and functional outcomes.^43–45^ Although IgGs such as trastuzumab contain 88 lysines (about 40 solvent-exposed under native conditions), experimental mapping studies report variable, often incomplete, and unpredictable site coverage,^46^ with broader site identification, e.g., 46 or up to 82 partially occupied sites,^44^ typically observed under more forcing or denaturing conditions. This variability reflects pronounced condition-dependent selectivity governed by the local lysine microenvironment,^47^ reaction parameters, and the specific reagents employed. For example, direct comparisons of NHS ester and formaldehyde-based modification further demonstrate markedly different site distributions, underscoring the limited predictive value of solvent accessibility or structural models alone.^48^ While recent studies have begun to examine site prevalence and potential CDR involvement,^44,45^ systematic prevalence-ranked mapping under defined reagent conditions remains limited.

Therefore, to pinpoint the conjugation sites (Table S6), we further analyzed trastuzumab conjugates at DAR values of 3.7, 11.4, and 12.8, respectively, using nLC–MS/MS as a method for mapping (Figure 5c, i–iii). To this end, we developed an analytical workflow enabling detailed peptide-level conjugation-site mapping from minimal antibody quantities (0.5 μg), employing an elevated trypsin-to-protein ratio (1:1) and nLC–MS/MS analysis under controlled conditions. This reduced-input approach contrasts with earlier multilevel characterization studies of trastuzumab and other antibodies typically performed at the milligram scale,^49^ and complements more recent lysine-reactivity investigations and ADC mapping workflows that rely on higher material input,^50^ e.g., 30 μg^51^ (see Section 2.4.3. in Supporting Information). In each case, we identified the conjugation positions within the heavy (H) and light (L) chains and then performed intersection statistical analysis to rank the conjugation sites from least (low) to most (high) probable (Figure 5c). Fifteen lysine residues (H43, H293, H65, H225, H249, L39, L145, H216, H217, H221, H323, H343, H417, L42, and L188) were identified as conjugation sites per individual light and heavy chain (30 total across the two identical light and heavy chains), with varying modification prevalence (Figure 5c, iv). One site (two per antibody) was located within the CDR region(s), but exhibited only moderate modification likelihood, suggesting that antigen binding could be largely preserved.

We further evaluated the prepared trastuzumab–carborane conjugates in cell-based assays to assess their functional activity (Figure 6). Notably, trastuzumab conjugates with DARs of 3.7 (37 boron atoms) and 9.5 (95 boron atoms) retained activity comparable to native trastuzumab in BT-474 cells at 50 nM (Figure 6a, for details see Supporting Information, Section 3.1). These results indicate that, even at relatively high DAR values, antigen recognition and downstream biological activity are largely preserved, highlighting the compatibility of carborane modification with antibody function.

**Figure 6:**
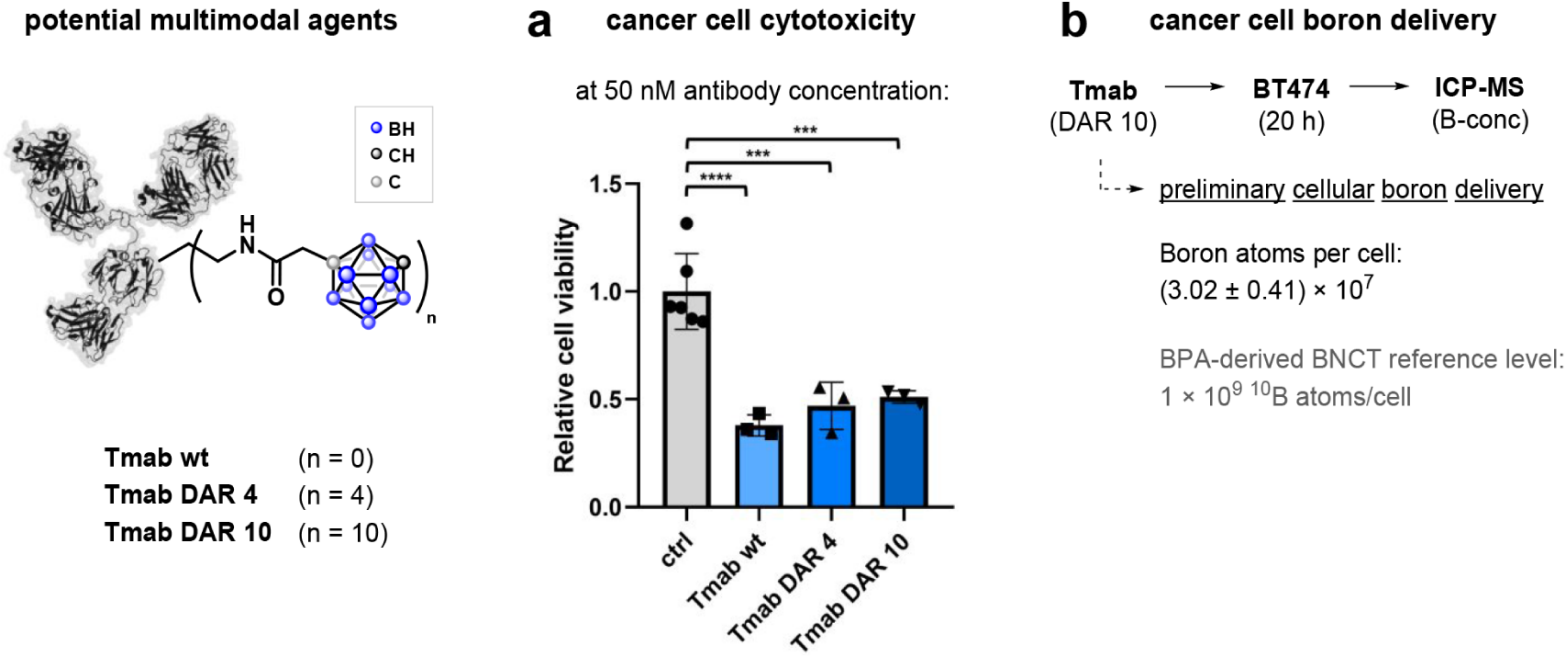
Carborane-conjugated trastuzumab constructs preserved wild-type cytotoxic activity in BT-474 cells while enabling delivery of relevant boron concentrations. (a) Comparable inhibition of cell proliferation was observed for both Tmab conjugates with DARs of 4 and 10, corresponding to approximately 40 and 100 bound boron atoms, respectively. BT-474 cell lines were incubated with the constructs at a concentration of 50 nM, and viability was assessed using the MTT assay. All experiments were conducted in triplicate, with error bars representing variability. Statistical significance was evaluated (ns *p* > 0.05; *p < 0.05; **p < 0.01; ***p < 0.001; ****p < 0.0001) using one-way ANOVA followed by Dunnett’s multiple comparisons test. (b) Workflow for assessing cell-associated boron delivery, including preliminary uptake data for boron-rich trastuzumab. BT-474 cells were treated with boron-rich trastuzumab (1 μM, DAR 10) for 20 h, extensively washed to remove nonassociated material, and digested prior to ICP-MS analysis of cell-associated boron. Data are presented as mean ± SD from two independent measurements (*n* = 2), with SD calculated as the sample standard deviation. Supernatants and control samples were below the limit of detection (LOD). The indicated value represents a boron concentration relevant in the context of BNCT.

Encouraged by the preserved cytotoxic activity of the boron-rich conjugates relative to the parent antibody, we preliminarily evaluated their ability to deliver boron to cancer cells. To this end, BT474 cells (1 × 10^6^ cells per well) were incubated with 1 μM of DAR 10 trastuzumab conjugate for 20 h, followed by extensive PBS washing and acid digestion of the remaining adherent cells prior to ICP-MS analysis (Figure 6b). An average boron concentration of 0.163 ± 0.022 μg/L per well was determined, corresponding to at least (3.02 ± 0.41) × 10^7^ boron atoms per cell, while controls and final wash supernatants remained below the limit of detection (LOD = 0.04 μg/L), confirming that the detected boron was cell-associated (for details see Supporting Information, Section 3.2.) Although approximately 30-fold lower than the commonly cited 1 × 10^9 10^B atoms per cell benchmark derived from BPA-based BNCT studies, antibody-mediated internalization proceeds via a fundamentally different biological pathway than diffusion-driven small-molecule uptake. Accordingly, the distinct cellular delivery and localization properties of antibody-mediated boron transport may enable therapeutically relevant effects at boron concentrations lower than those typically reported for BPA, thereby supporting further optimization of this targeted delivery strategy. Incidentally, the actual cellular boron concentrations may be even higher, as ICP-MS^52^ may underestimate boron levels due to incomplete quantitative recovery, further highlighting the promise of this approach.

Combining the therapeutic profile of the antibody with its simultaneous ability to act as a boron delivery agent for subsequent BNCT could establish such structures as multimodal agents for cancer treatment.

## Conclusion

We establish carborane–NHS esters as a versatile platform for amine-targeted functionalization, enabling rapid and efficient preparation of small-molecule, peptide, and antibody–carborane conjugates. The bifunctional design of carborane–NHS esters allows both intramolecular linkages and intermolecular couplings, the latter being tunable toward polymer formation, as demonstrated in the preparation of first-in-class peptide–carborane polymers. Among other applications, these structures have potential for BNCT, combining efficient (high) boron incorporation with maintained aqueous solubility through their tunable architecture. Importantly, the lysine conjugation strategy proved highly effective for generating antibody conjugates with high boron loadings (DARs up to 12.8, corresponding to 128 boron atoms per antibody). The reported analytical workflow enables detailed peptide-level mapping from low amounts of antibody using a high trypsin-to-protein ratio and controlled nLC–MS/MS, achieving comprehensive site-specific conjugation analysis. By combining reduced-input nLC–MS/MS analysis with prevalence-ranked site mapping, this work provides experimentally validated insight into native IgG lysine reactivity toward NHS reagents bearing hydrophobic (carborane) substituents, delineating site susceptibility under defined, native-like conjugation conditions. Trastuzumab conjugates with DARs of 4 and 10 were evaluated *in vitro* in cell-based assays and retained bioactivity comparable to the parent antibody, underscoring the compatibility of this modification with antibody function. Furthermore, preliminary cellular boron-delivery studies with the boron-rich antibody conjugate demonstrate boron concentrations within a potentially BNCT-relevant range. Considering its mechanism of action differs fundamentally from that of the archetypal BPA, these results highlight the promise of this approach for further development. Together, these findings highlight carborane–NHS esters as a powerful tool for constructing multimodal bioconjugates with potential utility as targeted boron delivery agents in anticancer therapy, combining established antibody mechanisms with boron delivery for BNCT.

## Supplementary Material



## Notes

Additional experimental details and characterization data including ^1^H, ^11^B, and ^13^C NMR, IR spectra, and mass spectra (PDF)
